# A68 THERAPEUTIC EFFECTS OF MEDICINAL CANNABINOIDS ON THE GASTROINTESTINAL SYSTEM IN PEDIATRIC PATIENTS: A SYSTEMATIC REVIEW

**DOI:** 10.1093/jcag/gwab049.067

**Published:** 2022-02-21

**Authors:** M Sunil, P Karimi, R Leong, G Zuniga-Villanueva, E Ratcliffe

**Affiliations:** 1 Medical Science, Pediatrics, McMaster University Faculty of Health Sciences, Hamilton, ON, Canada; 2 McMaster University, Hamilton, ON, Canada; 3 McMaster University Faculty of Health Sciences, Hamilton, ON, Canada; 4 Pediatrics, McMaster University, Hamilton, ON, Canada

## Abstract

**Background:**

Changes in cannabis legalization in recent years have led to an increasing interest in medicinal cannabinoids for a variety of therapeutic uses, including those which target the gastrointestinal tract. These effects are mediated by interactions with the endocannabinoid system. As this system is present in early life, it is important to ensure pediatric representation in clinical studies regarding cannabinoid use.

**Aims:**

We conducted a systematic review to assess the efficacy of medicinal cannabinoids in treating gastrointestinal symptoms in pediatric patients.

**Methods:**

A literature search of Medline, Embase, CINAHL, Web of Science and the Cochrane Library was conducted from inception. Study design, patient characteristics, type, dose and duration of medicinal cannabinoid therapy, and gastrointestinal outcomes were extracted.

**Results:**

From 7,303 records identified, five studies met all inclusion criteria. The focus of the included studies ranged from chemotherapy-induced nausea, inflammatory bowel disease and gastrointestinal symptoms associated with severe complex motor disorders. Results varied based on the symptom being treated, the type of cannabinoid, and the patient population, however, the most consistently improved symptom was chemotherapy-induced nausea.

**Conclusions:**

Medicinal cannabinoids may have a potential role in treating specific gastrointestinal symptoms in specific patient populations. The limited number and heterogenicity of included studies highlight the requirement for future research to distinguish the effects amongst different cannabinoid types, patient populations and examine drug-interactions. In addition, the molecular interplay between disease processes, the endocannabinoid system and medicinal cannabinoids require investigation to further understand the mechanisms behind this intervention.

**Funding Agencies:**

Natural Sciences and Engineering Research Council of Canada; Farncombe Family Digestive Health Research Institute

